# Ultra-High Strength in FCC+BCC High-Entropy Alloy via Different Gradual Morphology

**DOI:** 10.3390/ma17184535

**Published:** 2024-09-15

**Authors:** Ziheng Ding, Chaogang Ding, Zhiqin Yang, Hao Zhang, Fanghui Wang, Hushan Li, Jie Xu, Debin Shan, Bin Guo

**Affiliations:** 1 Key Laboratory of Micro-Systems and Micro-Structures Manufacturing of Ministry of Education, Harbin Institute of Technology, Harbin 150001, China; 17805602723@163.com (Z.D.); wangfanghui1014@163.com (F.W.); hushan1124@163.com (H.L.); shandb@hit.edu.cn (D.S.); bguo@hit.edu.cn (B.G.); 2 National Key Laboratory for Precision Hot Processing of Metals, Harbin Institute of Technology, Harbin 150001, China; zhiqinyang@foxmail.com; 3 School of Materials Science and Engineering, Harbin Institute of Technology, Harbin 150001, China; haozhang_1838@163.com; 4 Department of Materials Science and Engineering, Pohang University of Science and Technology, Pohang 37673, Republic of Korea

**Keywords:** dual-phase high-entropy alloys, high-pressure torsion, microstructure, mechanical properties

## Abstract

In this study, high-pressure torsion (HPT) processing is applied to the as-cast Al_0.5_CoCrFeNi high-entropy alloy (HEA) for 1, 3, and 5 turns. Microstructural observations reveal a significant refinement of the second phase after HPT processing. This refinement effect is influenced by the number of processing turns and the distance of the processing position from the center. As the number of processing turns or the distance of the processing position from the center increases, the fragmentation effect on the second phase becomes more pronounced. The hardness of the alloy is greatly enhanced after HPT processing, but there is an upper limit to this enhancement. After increasing the number of processing turns to 5, the increase in hardness at the edge becomes less significant, while the overall hardness becomes more uniform. Additionally, the strength of the processed alloy is significantly enhanced, while its ductility undergoes a noticeable decrease. With an increase in the number of processing turns, the second phase is further refined, resulting in improvement of strength and ductility.

## 1. Introduction

High-entropy alloys (HEAs) are those that contain at least five principal elements [1]. The highly uniform distribution of chemical composition endows HEAs with outstanding properties, making them a popular research topic in recent years [2,3,4,5]. As research on HEAs progresses, the relationship between mechanical properties and microstructure of HEAs has attracted increasing attention [6,7,8,9]. Compared to traditional single-phase HEAs, dual-phase HEAs simultaneously contain two different crystal phases: a face-centered cubic (FCC) phase and a body-centered cubic (BCC) phase. A complex reticular structure is formed by these different crystal phases in the alloy, resulting in excellent mechanical properties [10,11,12]. Dual-phase HEAs may offer a solution to overcome the strength–ductility trade-off by deliberately exploiting their heterophase nature to achieve superior mechanical properties. The presence of heterointerfaces in dual-phase HEA microstructures forms barriers for slip, resulting in higher strength than conventional single-phase HEAs. For instance, as-cast Al_0.5_CoCrFeNi alloy with a duplex FCC+BCC microstructure exhibits exceptional strain hardening rates of 6 GPa at high strains (>30%) at room and cryogenic temperatures, which was attributed to the formation of deformation twinning in the FCC phase [13]. The demand for improved damage tolerance has pushed scientific research towards the development of new materials possessing both high strength and high ductility. Therefore, dual-phase high-entropy alloys that can meet these demands are highly worthy of in-depth study.

The AlxCoCrFeNi system is currently one of the most studied HEA systems [14,15,16,17]. When x ≤ 0.4, the alloy is a single-FCC-phase alloy. When x reaches 0.5, the BCC phase begins to appear in the alloy, resulting in differences in the mechanical properties of the alloy. J. Joseph [18] investigated the influence of Al content on the microstructure and mechanical properties of the alloy, finding that as the Al content increased, the BCC phase content of the alloy gradually increased, resulting in increased strength but decreased ductility. Generally, the FCC phase exhibits high ductility and low strength, while the BCC phase exhibits the opposite, being brittle and hard. The composition and structure of these two phases have a significant impact on the alloy’s mechanical properties. Alloys with lower BCC phase content can have higher strength while maintaining a certain degree of plasticity. Al_0.5_CoCrFeNi is composed of certain BCC and FCC phases. It utilizes the high strength of the BCC phase and the good plasticity of the FCC phase, with a good balance of mechanical properties, and is widely used in aerospace, automotive and other fields. Aizenshtein et al. [19] investigated the microstructure, kinetics, and thermodynamics of Al_0.5_CoCrFeNi at T ≥ 800 °C. They not only proved that the content of the two phases in the alloy is related to temperature but also found that deformation before heat treatment can change the BCC phase morphology, thereby effectively reducing the yield stress. Niu et al. [20] successfully fabricated nanostructured alloys through heat treatment approaches. By controlling the content of the BCC phase and the size of the precipitates, the yield strength and elongation, respectively, reached 834 MPa and 25%. The microstructure of the alloy, including the second-phase size, also greatly influences its mechanical properties [21,22,23]. Therefore, studying how to adjust the microstructure of HEAs to achieve balanced and excellent mechanical properties is an important research direction.

In recent years, several plastic deformation processes such as equal-channel angular pressing (ECAP) and high-pressure torsion (HPT) have received considerable attention [24,25,26,27,28,29]. Compared to several other plastic deformation processes, HPT has the advantages of simple operation and the ability to introduce controlled plastic strain. In terms of microstructure control, HPT can achieve second-phase fragmentation and refinement by applying shear strain to the material, thereby improving its mechanical properties [30,31,32]. Zheng et al. [33] conducted HPT on FeNiCoCu high-entropy alloy (HEA) and (FeNiCoCu)_86_Ti_7_Al_7_ HEA with up to 10 turns. Microstructural observations and mechanical property tests showed significant second-phase refinement and strength enhancement in both alloys after HPT treatment. Hyogeon Kim [34] studied the mechanical properties and microstructural evolution of 7075 aluminum alloy processed by high-pressure torsion, indicating significant enhancement in the mechanical properties of nanocrystalline materials due to microstructural changes in high-temperature plastic deformation metal alloys.

Based on recent research of dual-phase HEAs and high-pressure torsion, this study subjected Al_0.5_CoCrFeNi cast alloy to high-pressure torsion processing with different numbers of turns, investigating the influence of HPT processing turns on the microstructure and mechanical properties of the alloy and providing a detailed analysis of the strengthening effect of high-pressure torsion on the alloy. Due to the uneven shear strain introduced by HPT processing, the microstructure exhibited varying degrees of refinement, presenting a gradient structure. Mechanical performance testing showed that the strength and hardness of the alloy after HPT processing had been greatly improved, with a tensile strength increase of about 4.6 times and a hardness increase of about 1.5 times compared to the cast alloy. Meanwhile, the processed alloy still retained a certain degree of plasticity. The ultra-high strength brought about by HPT processing provides a theoretical basis and practical basis for the widespread engineering application of Al_0.5_CoCrFeNi EHEAs. This article first introduces the materials and experimental methods, then it analyzes the experimental data results, and finally, it obtains three main conclusions based on the analysis.

## 2. Experimental Details

**Figure 1:**
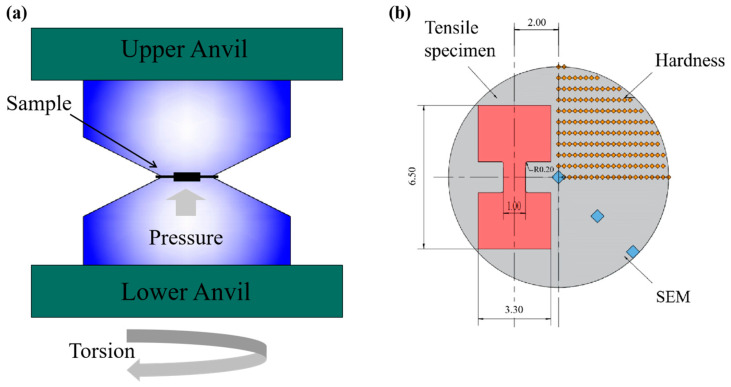
Schematic illustration of (**a**) HPT method and (**b**) sample and procedure used for different characterization methods.

A nominal composition Al_0.5_CoCrFeNi (in molar radio) high-entropy alloy (HEA) was produced by arc melting in an argon atmosphere using high-purity metals (more than 99.99 wt.%). The chemical composition of the Al_0.5_CoCrFeNi EHEAs is detailed in Table 1. To improve chemical homogeneity, the ingot was re-melted at least five times. The molten alloy was cast into a mold to obtain a cylindrical rod having dimensions of 100 mm in length and 20 mm in diameter. Samples having a diameter of 10 mm and a thickness of 1.5 mm were fabricated and then deformed though high-pressure torsion (HPT) at room temperature using a constrained HPT apparatus. The high-pressure torsion apparatus consists of a press and a torsion mold. While the press applies pressure in the height direction of the deformed body, it also applies a torque on its cross-section through active friction, causing the deformed body to undergo plastic deformation of axial compression and tangential shear deformation. The torsion mold is shown in Figure 1a. The pressures and the rotation speeds were construed at 6 GPa and 1 rpm, respectively. The HPT turn was set as 1, 3, and 5, and five HPT samples was repeated for each turn.

In order to accurately assess the influence of different HPT processing turns on hardness of surface, Vickers hardness testing was carried out via a semi-automatic Vickers hardness tester with 200 g loading force and 10 s holding. A quarter-circle area was selected with a transverse point spacing and rows spacing of 0.25 mm and 0.5 mm, which means that 200 points in total were measured.

The tensile properties of the specimens for different HPT processes were performed at room temperature with a strain rate of 0.001 s^−1^ using an AG-X100kN universal testing machine (Shimadzu Corporation, Kyoto, Japan). The dog-bone-shaped specimens were designed with a gauge length of 2 mm and width of 1 mm [29]. In order to avoid the influence of the processing process on the structure or composition of the specimen, the tensile specimens were cut using low-speed WEDM. In order to obtain more accurate measurement results, digital image correlation (DIC) technology was employed for strain measurement. Before tensile testing, the surface of the tensile specimens was polished and the surface speckles were created using ink powder. Each tensile experiment was replicated four times to mitigate experimental variability, thereby enhancing the scientific validity and precision of the experiments.

X-ray diffraction (XRD) was carried out on the obtained HPT disk-shaped specimens to determine the phase structure by comparing the diffraction pattern of the sample with the diffraction pattern in the standard database and finding the pattern with the highest matching degree. The operating parameters were 40 kV and 30 mA in the 2θ range of 20° to 100°, with a scanning step of 0.01° and a scanning speed of 2°/min. Microstructural characterization was performed employing scanning electron microscopy (SEM). The equipment used was a Zeiss Gemini-560 field emission scanning electron microscope (Carl Zeiss AG, Oberkochen, Germany) with an acceleration voltage of 25 kV. According to the different deformations in each turn, the specimens were divided into the central region, half-radius region, and edge region. SEM was used to capture the microstructure of the fracture morphology in order to better characterize the tensile properties of the material after the tensile tests.

## 3. Results and Discussion

### 3.1. Microstructure Evolution of Al_0.5_CoCrFeNi High-Entropy Alloy

#### 3.1.1. XRD Analysis

**Figure 2:**
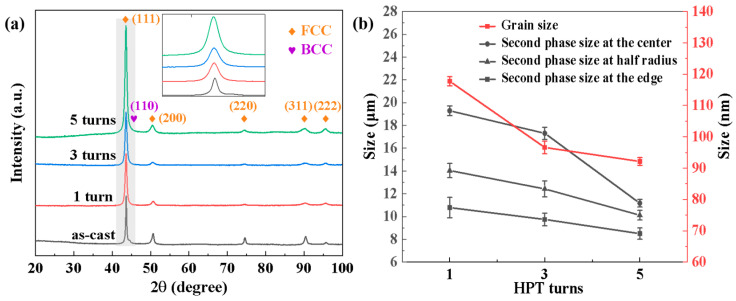
(**a**) XRD analysis for the as-cast sample and after HPT through 1, 3, and 5 turns; (**b**) grain size and second-phase size under different processing cycles.

The XRD scan results in Figure 2a reveal that the initial cast alloy exhibited the structure of FCC and BCC phases, consistent with the SEM image results. With an increase in the number of HPT processing cycles, the intensity of the XRD diffraction peaks underwent a two-stage variation. In the first stage, applying high-pressure torsion deformation led to a decrease in the diffraction peak intensity, but the decline was limited to only 4.3%. In the second stage, as the number of processing cycles increased, the diffraction peak intensity rose significantly. Compared to when the number of processing turns was 3, the strength increased by 70.9% when the number of processing turns was 5. Similar phenomena were observed by P. F. Yu et al. [35] when studying the effects of high-pressure torsion on Al_0.1_CoCrFeNi high-entropy alloys. As their research has shown, equivalent strain, $\varepsilon_{eq}$, during HPT is calculated using the following equation:

(1) $$\varepsilon_{eq}=\frac{2\pi rN}{\sqrt{3}h}$$

where *N* represents the number of rotations in HPT, *r* denotes the distance from the center of the circular sample, and *h* stands for the sample thickness. As indicated by Formula (1), significant shear strain is applied to the material during HPT processing, in a manner proportional to the number of torsion cycles. In the first stage, as the number of processing cycles is small, the shear strain generated by processing leads to an increase in lattice strain, fragmentation, and rearrangement of grains, resulting in the loss of crystalline perfection and a decrease in X-ray scattering, which causes the reduction in diffraction peak intensity. The variation of XRD peak intensities is similar to that caused by the addition of multi-principal elements with different atomic sizes [36]. In the second stage, as the number of processing cycles increases, the shear strain acting on the alloy becomes large enough to cause the grains to be fully broken and form a more uniform microstructure, thereby reducing the scattering effect of differently oriented grains on X-rays and increasing the intensity of XRD diffraction peaks [37]. During the high-pressure torsion processing, the peak width of the XRD gradually broadens, especially for the (111) plane. The FWHM values at different cycles of the diffraction peaks of the (111) plane were measured using Origin software (https://www.originlab.com/ (accessed on 12 September 2024)), and they were, respectively, 0.534, 0.579, and 0.602. It was demonstrated that as the number of processing turns increased, the FWHM values continued to increase and the peak width continued to widen. Figure 2b shows the changes in grain size and second-phase size under different processing turns. As the number of processing turns increased, the grain size decreased from 118 nm to 92 nm. The decrease in grain size will lead to a broadening of diffraction peak width, which was consistent with the XRD pattern. It is noteworthy that no distinct BCC phase peak was observed in the XRD results of the HPT-processed specimens. Xue et al. [38] found that the size of the phase was greatly decreased, due to the phenomenon of second-phase fragmentation refinement introduced by HPT processing. Additionally, it may also be due to grain boundary slip, causing the small grains of the second phase to fracture, leading to thin layers on the grain boundaries of the primary phase [39]. Consequently, the characteristic peaks of the second phase are undetectable once grain size reaches a certain threshold. Zhang et al. [40] also found, in the CoCrNiMo high-entropy alloy, that although the second phase can be observed via SEM, it cannot be detected by XRD due to the small content of the second phase. As described in the subsequent scanning images, the alloy remains as FCC+BCC phases after HPT processing, indicating that high-pressure torsion processing did not alter the alloy phase structure.

#### 3.1.2. SEM-BSE Images of Al_0.5_CoCrFeNi High-Entropy Alloy

**Figure 3:**
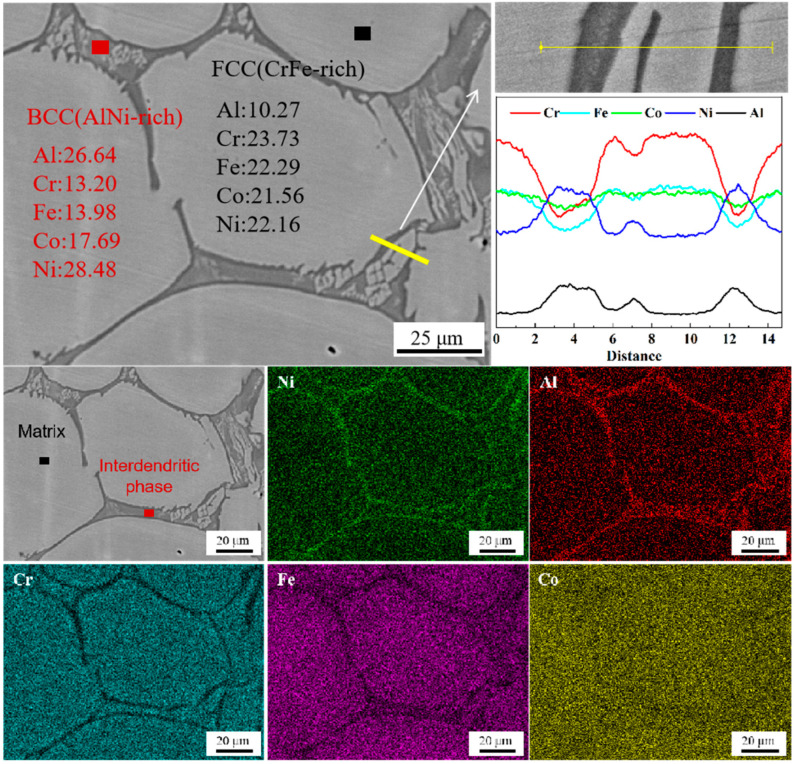
Microstructure and element distribution map of Al_0.5_CoCrFeNi as-cast alloy.

Figure 3 shows the microstructure of the Al_0.5_CoCrFeNi as-cast specimen. The initial cast alloy exhibited a typical dendritic structure, with the matrix phase being the face-centered cubic (FCC) phase and the inter-dendritic second phase being the body-centered cubic (BCC) phase. The element distribution map and the results of the lines scanning pointed by the arrows in Figure 3 also indicates an enrichment of the Cr and Fe elements in the matrix phase and a lack of the Al and Ni elements. Conversely, the inter-dendritic region exhibits the opposite elemental distribution. The Co elements are evenly distributed in both phases. This result is in accord with other research findings on Al_0.5_CoCrFeNi high-entropy alloys [41,42].

**Figure 4:**
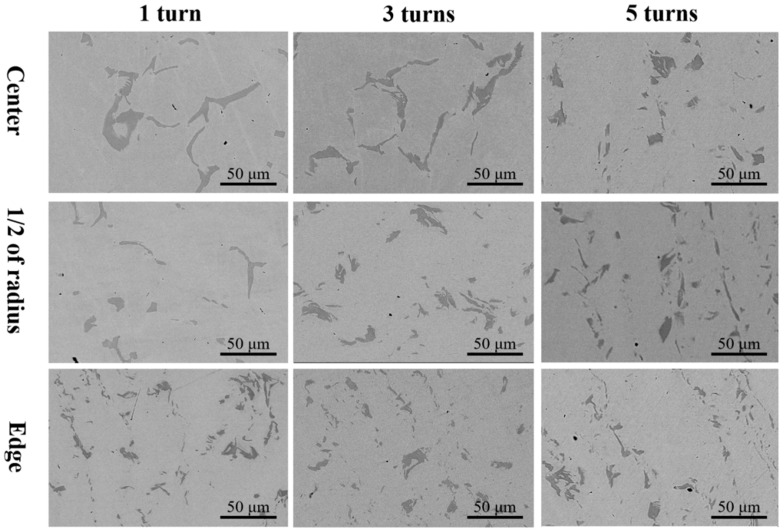
SEM-BSE images showing the microstructure at the center, 1/2 of radius, and edge region after HPT for 1, 3, and 5 turns.

Figure 4 displays the microstructure of different samples processed by 1, 3, and 5 torsion cycles. In comparison to the initial as-cast sample, the second phase was significantly fragmented after HPT processing. The initial inter-dendritic structure was disrupted, showing a trend of dispersed distribution. High shear strain was introduced during the HPT processing, resulting in the fragmentation and refinement of the second phase [30,31,32]. Different regions of the same HPT-processed sample also exhibited distinct microstructures. According to the vertical comparison shown in Figure 4, the level of refinement of the second phase was significantly higher at the edge region compared to the central. According to Formula (1), the magnitude of shear strain induced by HPT processing relates to the distance from the processing position to the center [43]. The closer to the center, the smaller the shear strain induced by HPT. The level of fragmentation and refinement of the second phase was notably higher at the edge compared to the central region. As is shown in Figure 2b, when the number of processing cycles is 1, the diameter of the second phase at the edge is 10.78 μm, and the diameter at the center is 19.28 μm, with a refinement of about 44%. Especially during the initial stages of HPT processing (e.g., at 1 cycle), some dendritic structures were still kept in the central region because of the lower shear strain. However, when the torsion turns reached 3 and 5, the difference in the level of refinement of the second phase between the edge and the 1/2 radius region was relatively small, which means that there is a limit to the refining effect of shear strain on the second phase. Due to the lower shear strain experienced, the level of refinement of the second phase in the central region was limited and gradually increased with the number of processing cycles [44]. After increasing the number of processing cycles, most of the dendritic structures were completely disrupted. Due to the strain induced by circumferential shear strain, the second phase was fragmented and tended to distribute along the rotational streamline direction.

**Figure 5:**
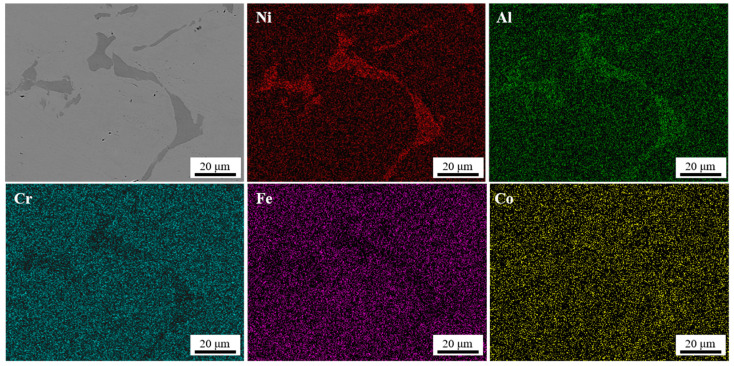
Element distribution map at the center after HPT for 1 turn.

Figure 5 shows the distribution of elements when the number of torsion cycles is 3. The application of high-pressure twisting processing did not cause a change in the chemical composition of the BCC and FCC phases but rather caused the second phase to break into smaller sizes, as reported earlier in other materials [45,46].

### 3.2. Mechanical Properties

#### 3.2.1. Hardness Evolution after HPT Processing

**Figure 6:**
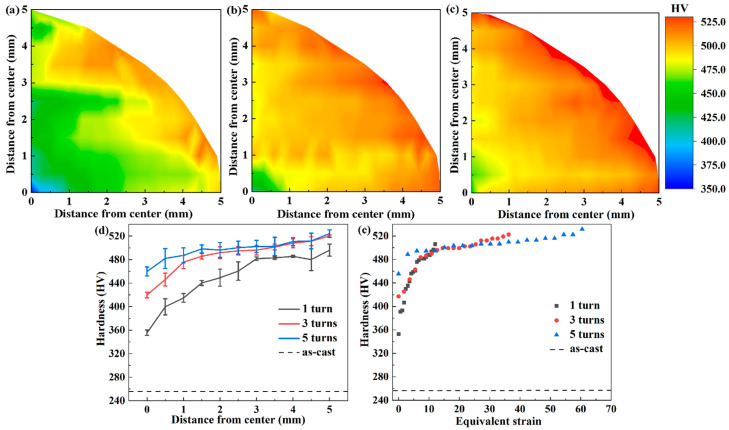
The Vickers microhardness plotted against (**a**) 0.5 turns, (**b**) 1 turn, (**c**) 3 turns, (**d**) distance from the disk center, and (**e**) equivalent strain.

Figure 6a–c illustrate the variation trend of Vickers microhardness along different positions of the Al_0.5_CoCrFeNi alloy under different HPT processing cycles, while Figure 6d displays the radial variation of Vickers microhardness. Due to the uniform surface hardness distribution of the initial as-cast samples, the hardness measurement method used for the HPT samples was not adopted. Instead, five random points were selected on the surface with a 1mm spacing between points. The test results show that the surface hardness of the as-cast sample was approximately 249 HV. Observing the test results in Figure 6, it is found that compared to the as-cast sample, the surface hardness was significantly increased after HPT processing. This is mainly due to the surface work hardening induced by HPT processing. When the number of torsion cycles was 1, the lowest surface hardness was 352 HV, which was about 30% higher than that of the as-cast sample. The surface hardness gradually increased with the increase of HPT processing cycles. When the number of torsion cycles rose to 3 and 5, the hardness reached a saturation level of around 530 HV. Further increasing the number of processing cycles did not significantly increase the hardness. Different shear strain was introduced into different regions during HPT processing. This resulted in lower hardness in the center of the sample and higher hardness at the edges when the number of processing cycles was small. After reaching saturation, continuing to increase the number of processing cycles introduced larger shear strain, which help to make the surface hardness of the sample more uniform. It could be observed that when the number of processing cycles was 1 and 3, the hardness in the center was about 140 HV lower than that at the edges; when the number of processing cycles was 5, the difference in hardness between the center and the edges decreased, which meant that the hardness in different regions became more uniform. The high shear and compressive stresses introduced by HPT processing generated a significant second-phase refinement [47,48]. This was the primary reason for the increase of hardness. This is also consistent with the results shown in Figure 2b. On the one hand, as the number of torsion cycles increased and the distance from the processing position to the center kept increasing, the grain size and second-phase size decreased, leading to an increase in hardness. On the other hand, after three cycles of processing, the refinement effect brought about by HPT was significantly weakened, resulting in only a smaller increase of hardness. When the processing position was at the edge, the size of the second phase did not change significantly when the number of processing cycles was increased, resulting in little change in hardness. When the processing position was located at the center of the circle, increasing the number of processing cycles could significantly refine the second phase and greatly increase hardness. Figure 6e shows the functional relationship between the equivalent strain introduced by HPT processing and hardness, where the equivalent strain was calculated by Formula (1) and was proportional to distance and number of torsion cycles. As the number of machining cycles or distance from the center increased, the hardness increased continuously until saturation.

According to previous research results [49], the hardness change after HPT processing can be classified into three types: no recovery type, with recovery type, and with softening type. The hardness changes of the Al_0.5_CoCrFeNi alloy after HPT processing conform to the no recovery type, where the Vickers microhardness increases with the increase of equivalent strain and reaches saturation with the increase of processing cycles, stabilizing thereafter. This type is typical for most high-entropy alloys [50,51,52].

#### 3.2.2. Tensile Properties

**Figure 7:**
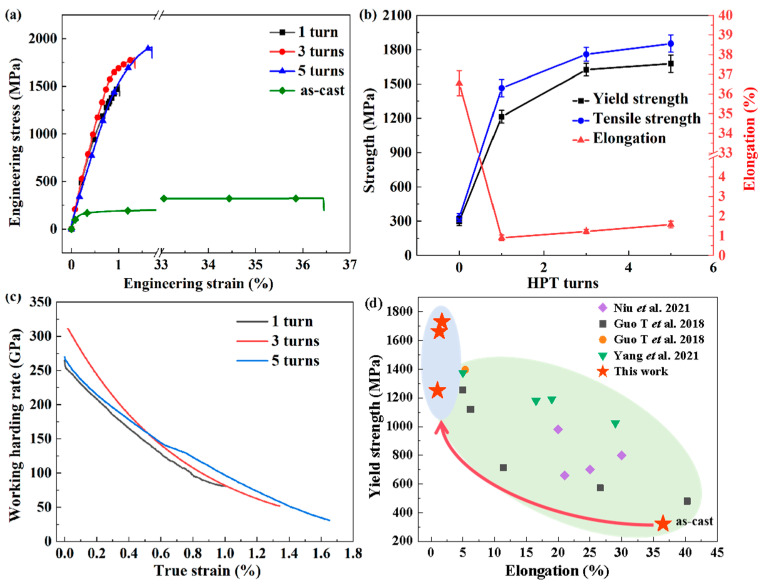
Tensile properties of Al_0.5_CoCrFeNi HEAs processed by HPT: (**a**) engineering stress–strain curves; (**b**) changing tendencies of yield strength, tensile strength, and elongation; (**c**) work hardening rate plotted against true strain; (**d**) comparison with the data collected from other studies [58,59,60].

To visually represent the trend of performance changes between the as-cast and HPT-processed specimens, Figure 7b compares the elongation, tensile strength, and yield strength. The as-cast Al_0.5_CoCrFeNi alloy exhibited a relatively high elongation of 36.5% and a lower strength of 323MPa. Compared to the as-cast specimens, the elongation of the HPT-processed specimens decreased significantly to around 4%, while the strength increased substantially. Consistent with the findings of most studies [53,54], on one hand, the HPT processing introduced a large number of dislocations and low-angle grain boundaries. During tensile deformation, dislocations became entangled and accumulated at grain boundaries, hindering the movement of grain boundaries and leading to the formation of numerous microcracks at grain boundaries, which resulted in lower elongation. On the other hand, the HPT processing promoted a more uniform distribution of the BCC and FCC phases and refined the BCC phase. These actions could suppress dislocation motion, leading to an increase in the alloy’s strength.

The engineering stress–strain curves of the HPT-processed specimens are depicted in Figure 7a. As the number of processing revolutions increased, both the strength and elongation of the specimens improved. Consistent with the results shown in Figure 2b, with the increase in the number of processing cycles, the shear strain on the sample increased, and the size of the second phase was further refined. As the size of the second phase gradually decreased, the strengthening effect of the second phase on the tensile strength of the alloy was further improved, resulting in a continuous increase in the tensile strength of the alloy with an increase in the number of machining cycles, which is consistent with previous research findings [55,56]. At the same time, as the number of processing revolutions increased, the second phase tended to be more thoroughly fragmented, leading to an increase in plasticity. Zheng et al. [33] conducted HPT on FeNiCoCu high-entropy alloy (HEA) and (FeNiCoCu)86Ti7Al7 HEA with up to 10 turns. They found that HPT processing can cause complex changes in the microstructure of alloys. Although the shear stress introduced by HPT processing generates some dislocations, it also leads to a decrease in the size of the second phase, thereby reducing the stress concentration level around it. Therefore, during the process of tensile deformation, severe stress concentration points will be delayed, thereby improving a certain degree of ductility. Meanwhile, it was observed that as the number of processing revolutions increases, the elongation gradually increases. This is contrary to the typical work hardening effect [57]. According to the study by Hyogeon Kim [34] on the influence of high-pressure torsion on Al7075 alloy, it was found that HPT processing not only provides large shear strain but also provides a large amount of frictional heat, which is conducive to generating complex microstructural changes. Large shear strain can lead to grain refinement, while the large amount of frictional heat generated by processing can cause the alloy to undergo certain dynamic recovery. The former is beneficial for improving the strength of the alloy, while the latter can lead to the disappearance of dislocations, resulting in a decrease in the work hardening rate of the alloy. Therefore, the samples having 3 processing turns exhibited higher working hardening rates than those having 5 processing turns during the tension process of the current study.

Figure 7d lists the elongation and tensile strength comparison of the Al_0.5_CoCrFeNi alloy under HPT processing and other processing methods [58,59,60]. It can be clearly seen that the alloy after HPT processing is located at the top left corner of the graph, demonstrating a significant increase in strength after HPT processing, far exceeding that of the alloy under other processing methods. Conversely, the alloy processed by HPT exhibited shortcomings in plasticity. We considered that there exists a clear trade-off relationship between strength and plasticity after HPT processing. However, according to the other literature, it is possible to perform heat treatment on the alloy after HPT processing to improve plasticity to a certain extent while sacrificing a certain level of strength, thus achieving a better combination of strength and plasticity [31]. These conjectures need further experimental investigation for verification.

#### 3.2.3. Fracture Analysis

**Figure 8:**
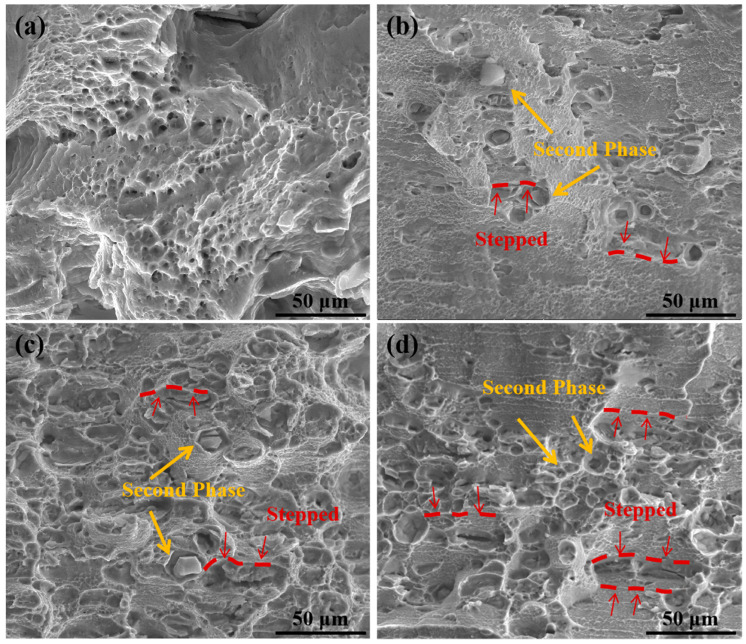
The microscopic morphology of fracture surface: (**a**) as-cast; (**b**) 1 turn; (**c**) 3 turns; (**d**) 5 turns.

Figure 8 shows the microstructure of the alloy’s tensile specimen after HPT processing. The fracture mode of the Al_0.5_CoCrFeNi alloy mainly involved tearing of the soft FCC phase, while the harder BCC phase particles remained in the matrix, which could be intuitively observed in the fracture morphology [61,62]. Near the BCC phase particles, a brittle fracture was observed, and the fracture surface appeared stepped, indicating the role of the second phase in hindering dislocation motion and reducing material plasticity. Consistent with the results shown in Figure 7b, when the number of processing revolutions was 1, the specimen exhibited low plasticity, with a brittle fracture and almost no observable dimples on the fracture surface. After increasing the number of processing revolutions to 3, the plasticity of the specimen improved, and the fracture surface showed a combination of dimples and river-like cleavage patterns. As the number of processing revolutions increases, a broken second phase can be observed in Figure 8. The second phase gradually refined, and its quantity increased. The hinderance effect of refined second phase on dislocations decreased, leading to an increase in the material’s ductility [30,31,32].

## 4. Conclusions

In this study, high-pressure torsion processing was applied to the as-cast Al_0.5_CoCrFeNi high-entropy alloy for 1, 3, and 5 turns, investigating its effects on the microstructure and mechanical properties of the alloy. The following conclusions were drawn:

1. The shear strain introduced by high-pressure torsion led to the refinement of the second phase, grain fragmentation, and rearrangement effects, without altering the alloy’s phase composition.
2. Significant enhancement in hardness was observed after high-pressure torsion processing of the Al_0.5_CoCrFeNi high-entropy alloy. The hardness gradually increased with the number of processing turns, and the hardness of various regions on the sample surface became progressively uniform. The hardness enhancement reached saturation, with a saturation hardness of approximately 530 HV, representing an increase of about 113% compared to the initial as-cast state.
3. The strength of the alloy was greatly improved after high-pressure torsion processing, increasing from 323 MPa to over 1500 MPa, but there was a decrease in ductility. The second phase was fragmented and refined by the introduction of shear strain, resulting in an increase in the ductility with an increasing number of processing turns.

Overall, high-pressure torsion significantly affected the properties of the as-cast Al_0.5_CoCrFeNi high-entropy alloy, enhancing its hardness and strength while sacrificing some ductility.

## Figures and Tables

**Table 1:** Chemical composition of the experimental HEAs.

Element	Al	Co	Cr	Fe	Ni
at.%	11.21	22.19	22.24	22.23	22.13

## Data Availability

The original contributions presented in the study are included in the article; further inquiries can be directed to the corresponding authors.
